# Metabolic interference impairs influenza A virus replication by dampening vRNA synthesis

**DOI:** 10.1038/s44298-025-00090-4

**Published:** 2025-03-28

**Authors:** Jens Kleinehr, Chiara Robin Bojarzyn, Michael Schöfbänker, Katharina Daniel, Stephan Ludwig, Eike R. Hrincius

**Affiliations:** 1 Institute of Virology Muenster (IVM), University of Muenster, Von-Esmarch-Straße 56, Muenster, Germany; 2 Institute of Virology, Faculty of Medicine and University Hospital Cologne, University of Cologne, Cologne, Germany

**Keywords:** Influenza virus, Virus-host interactions

## Abstract

For replication, viruses exploit the host cell metabolism for biosynthesis of viral components. Recently, we could show that inhibition of glycolysis interfered with IAV replication by impairing the regulation of the viral polymerase as a transcriptase or replicase. Here, we investigated how IAV replication and polymerase regulation is influenced by other metabolic pathways which are directly or indirectly linked to glycolysis. Therefore, we inhibited glutaminolysis, fatty acid synthesis (FAS), oxidative phosphorylation (OXPHOS), and the pentose phosphate pathway (PPP). Inhibition of these metabolic pathways led to a significant reduction of viral titers. Furthermore, the inhibition of glutaminolysis, FAS and OXPHOS unbalanced the cellular glycolysis and respiration network leading to a prolonged phase of viral transcription while replication was strongly decreased. Our data indicate that affecting the cellular glycolysis and respiration balance impairs the dynamic regulation of the viral polymerase, resulting in reduced synthesis of viral genomic RNA and viral particles.

## Introduction

By causing annual epidemics and occasional pandemics, influenza A viruses (IAV) are among the most threatening respiratory pathogens for human health. Due to its high mutation rate, IAV quickly develops resistance against common pathogen-targeted antivirals^1^. Therefore, the development of host-targeted antivirals, which bear a lower risk of emerging resistances, is gaining more attention. One potential host-targeted approach is metabolic interference, the partial pharmacological inhibition of metabolic pathways in order to restrict the spread of a pathogen. As intracellular pathogens without an endogenous intrinsic metabolism, viruses fully depend on the host metabolism and even modify it during an infection^2^. Upon influenza virus infection of cells, altered pathway activity or metabolite concentrations were described for glycolysis^3–5^, glutaminolysis^5,6^, fatty acid synthesis (FAS)^5–7^, oxidative phosphorylation (OXPHOS)^5,8^, the pentose phosphate pathway (PPP)^4,5^ and others. Enhanced activity of these pathways suggests their importance for IAV replication and spread. In addition, influenza virus infection deregulates the host energy metabolism in mouse models^9^. Recently, we have shown that pharmacological inhibition of glycolysis severely reduced viral replication by affecting the viral polymerase^10^. The treatment triggered a prolonged phase of viral transcription while the synthesis of viral genomic RNA (vRNA), which is conveyed by the same polymerase, was decreased. This observation suggests that there is a specific regulatory effect on the IAV polymerase rather than a general impact caused by reduced pools of metabolites such as ATP. Expanding our perspective, we investigated whether deregulation of the viral polymerase could be a common consequence of various metabolic interference approaches due to overlapping metabolic alterations within the infected cells. Hence, in this study we analyzed viral propagation and viral polymerase activity after treatment with inhibitors of glutaminolysis, FAS, OXPHOS and the PPP by using bis-2-(5-phenylacetamido-1,3,4-thiadiazol-2-yl)ethyl sulfide (BPTES), 5--(tetradecyloxy)-2-furancarboxylic acid (TOFA), oligomycin A and 6-aminonicotinamide (6-AN), respectively (Supplementary Fig. S1). As mentioned, all these pathways were previously described to be altered upon IAV infection and are directly or indirectly linked to glycolysis and to each other.

In this report, we demonstrate antiviral efficiency and safety of these inhibitors in vitro and shed light on a largely general effect of metabolic interference on the function of the viral polymerase and, consequently, on the life cycle of IAV.

## Results

### Metabolic interference severely decreases viral replication and production of viral progeny

**Fig. 1:**
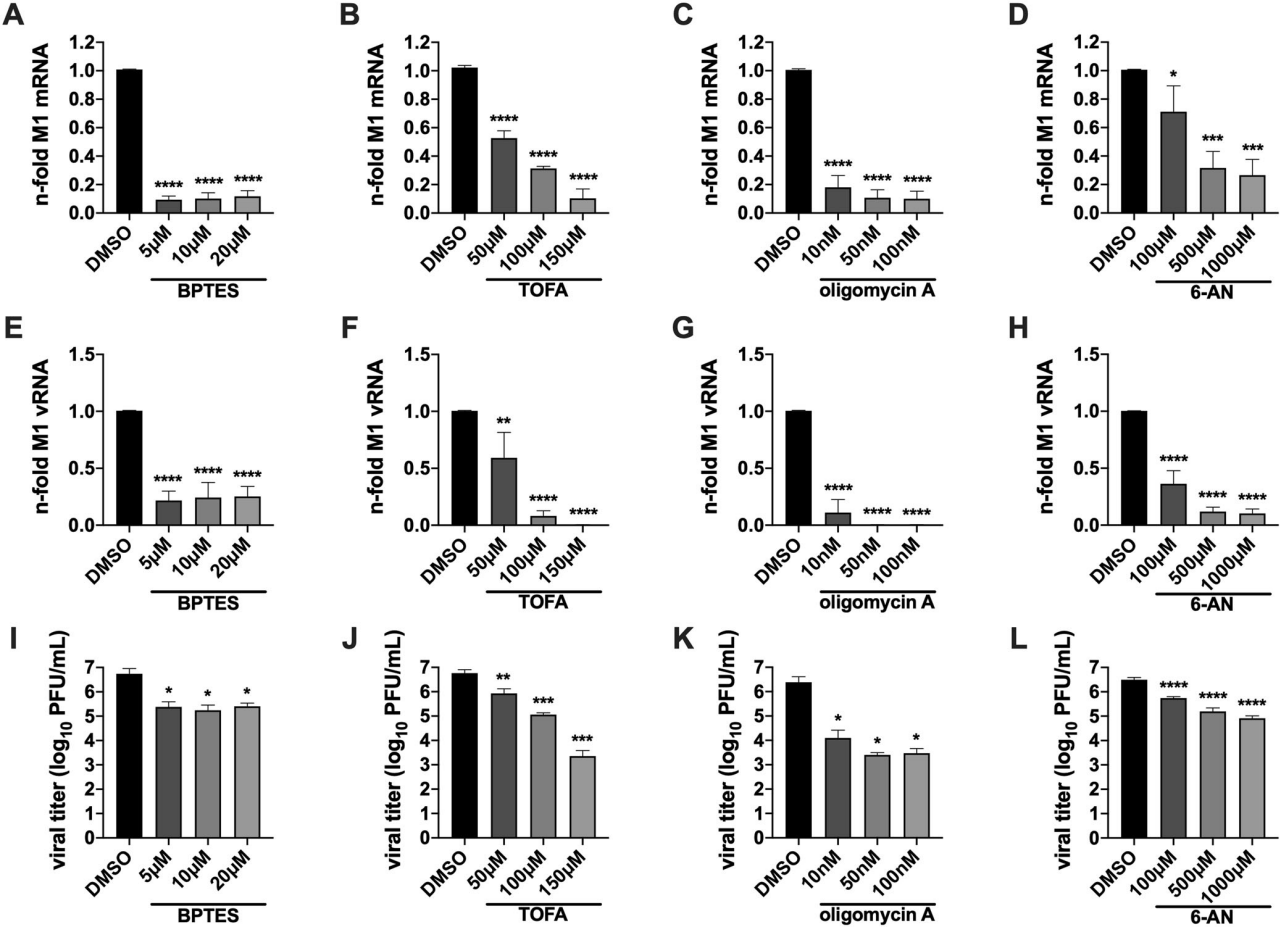
Metabolic interference inhibits viral RNA accumulation and virion production. A549 cells were infected with SC35M at an MOI of 0.001 for 30 min. Subsequently, the infected cells were treated with the indicated concentrations of the inhibitors for a total of 24 h since the beginning of the infection. **A**–**H** Afterwards, cells were lysed, total RNA was isolated, and cDNA was synthesized using either (**A**–**D**) an oligo(dT) primer to transcribe mRNA or (**E**–**H**) a fluA uni12 primer to transcribe vRNA. **A**–**H** Realtime qPCR was performed with technical duplicates, and the n-folds were calculated in reference to the DMSO control. **I**–**L** 24 hpi supernatants were collected and used to determine viral titers via plaque assay. **A**–**L** Depicted are the means ± SD of three independent experiments with biological triplicates per condition and experiment. Statistical significances were determined via ordinary one-way ANOVA with Dunnett’s correction, comparing all treated samples to their DMSO control. *P*-values are indicated as follows: <0.05 = *, <0.01 = **, <0.001 = ***, <0.0001 = ****.

Our first aim was to clarify whether the inhibition of glutaminolysis with BPTES, FAS with TOFA, OXPHOS with oligomycin A and the PPP with 6-AN restricts synthesis of IAV components and consequently would impair production of new viral particles. Hence, we analyzed the accumulation of viral transcripts (mRNA of matrix protein 1 (M1)), various viral proteins (polymerase acidic protein (PA), M1, nucleoprotein (NP) and non-structural protein 1 (NS1)), viral genome copies (vRNA of M1) and the amount of progeny virus 24 h post infection (hpi) of A549 cells with the IAV strain A/Seal/Massachusetts/1/80 H7N7 (SC35M) at a multiplicity of infection (MOI) of 0.001. We observed that under the influence of each metabolic inhibitor, the accumulation of viral mRNA (Fig. 1A–D) and viral genomic RNA (vRNA) (Fig. 1E–H) was severely decreased, with some differences in the level of reduction between the inhibitors. In line with these observations, viral titers were significantly reduced in the presence of each inhibitor (Fig. 1I–L).

**Fig. 2:**
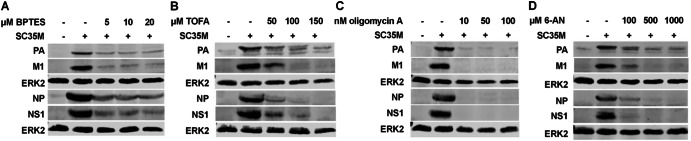
Metabolic interference results in a strong decrease of viral protein accumulation. **A–D** A549 cells were infected with SC35M at an MOI of 0.001 for 30 min. Subsequently, the infected cells were treated with the indicated concentrations of the inhibitors for a total of 24 h since the beginning of the infection. Then, cells were lysed, and proteins were separated via SDS-PAGE. Visualization of protein bands was done using primary antibodies binding PA (rabbit), M1 (mouse), NP (rabbit), NS1 (rabbit) and the loading control ERK2 (rabbit) and fluorescent-labeled anti-mouse (donkey) and anti-rabbit (donkey) secondary antibodies. Illustrated are exemplary protein bands from three independent experiments.

In line with the effects on viral mRNA synthesis, we observed that the accumulation of various viral proteins was also notably diminished (Fig. 2).

Hence, each inhibitor displayed a clear virus-restricting effect over a treatment period of 24 h. Within the concentration ranges used, TOFA and 6-AN exhibited clear dose-dependent effects, while the inhibitory activity of BPTES and oligomycin A on viral synthesis seemed to be quickly saturated at certain concentrations.

**Fig. 3:**
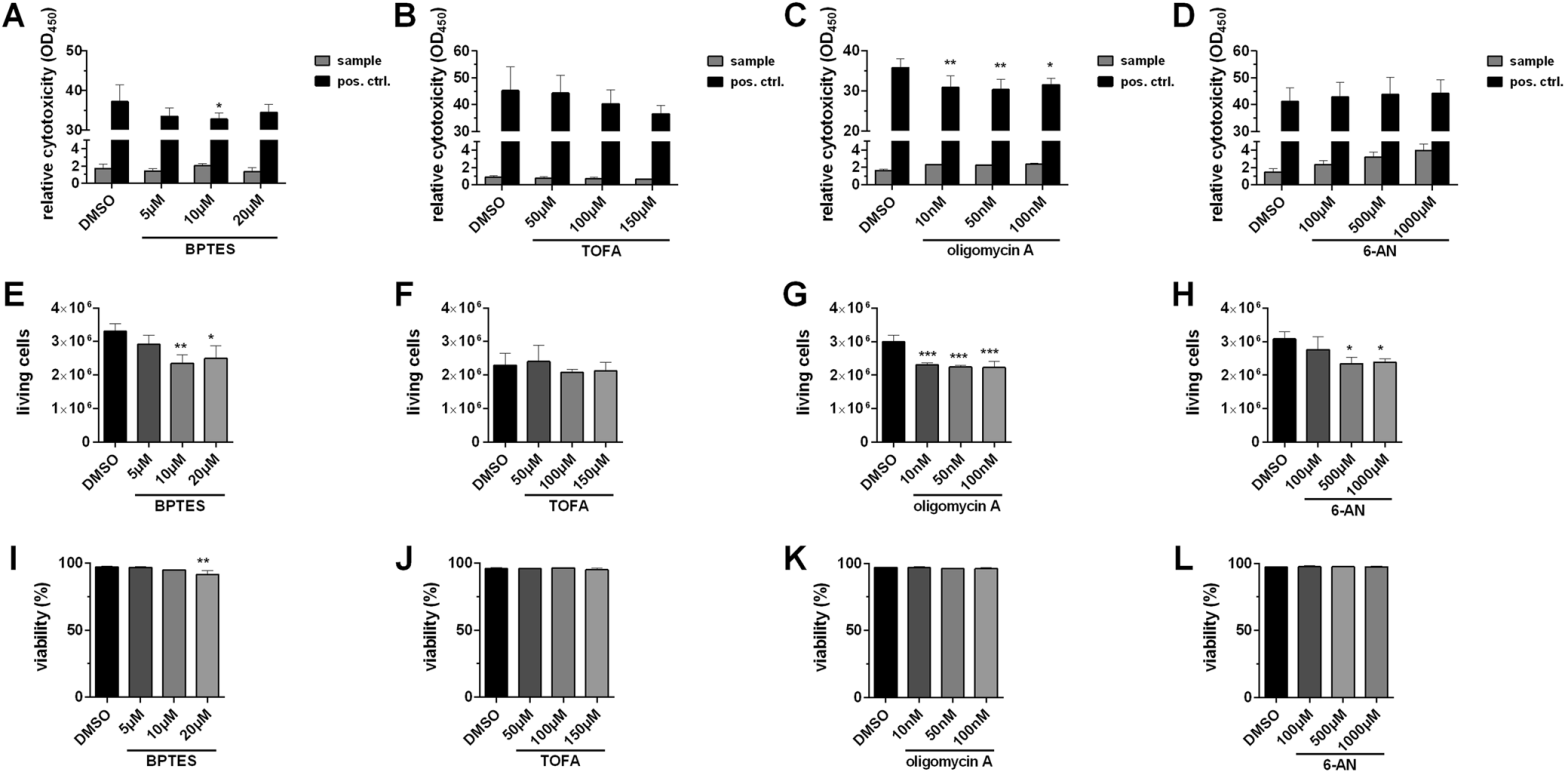
Metabolic interference exhibits barely cytotoxic but slight cytostatic effects. Uninfected A549 cells were treated with the indicated inhibitor concentrations for 24 h. **A**–**D** Afterwards, LDH assays were performed to determine the relative cytotoxicity of each treatment, or (**E**–**L**) cells were detached to determine the number of living and the viability through trypan blue exclusion. Depicted are the means ± SD of three independent experiments with biological triplicates per condition and experiment. Statistical significances were determined via (**A**–**D**) ordinary two-way ANOVA with Dunnett’s correction, comparing all treated samples of a group to their respective DMSO control or via (**E**–**L**) ordinary one-way ANOVA with Dunnett’s correction, comparing all treated samples to their DMSO control. *P*-values are indicated as follows: <0.05 = *, <0.01 = **, <0.001 = ***, <0.0001 = ****.

To confirm the absence of cytotoxic effects of the four inhibitors, we performed lactate dehydrogenase assays. In the observation period of 24 h we barely detected any cytotoxicity of the inhibitors in the concentration ranges used (Fig. 3A–D). However, trypan blue exclusion assays performed 24 h after treatment suggested a moderate cytostatic effect for at least BPTES, oligomycin A and 6-AN, which we concluded from decreased numbers of proliferating cells (Fig. 3E–H) next to a largely unaffected rate of viability of the same cells (Fig. 3I–L).

Taken together, within the applied concentration ranges, treatment with all four inhibitors resulted in a significant impairment of IAV propagation in the absence of considerable cytotoxic or, if any, only moderate cytostatic effects.

### Metabolic interference affects the viral life cycle mainly by impairing vRNA synthesis

After observing the cumulative impact of different modes of metabolic interference over a 24 h period, which encompassed multiple cycles of IAV replication, we became curious about their effects on a single viral life cycle lasting ~8 h. Consequently, we replicated the experiments using a higher MOI and incubated the infected cells with the inhibitors for 8 h only. Subsequently, we analyzed accumulation of viral mRNA, proteins, and vRNA.

**Fig. 4:**
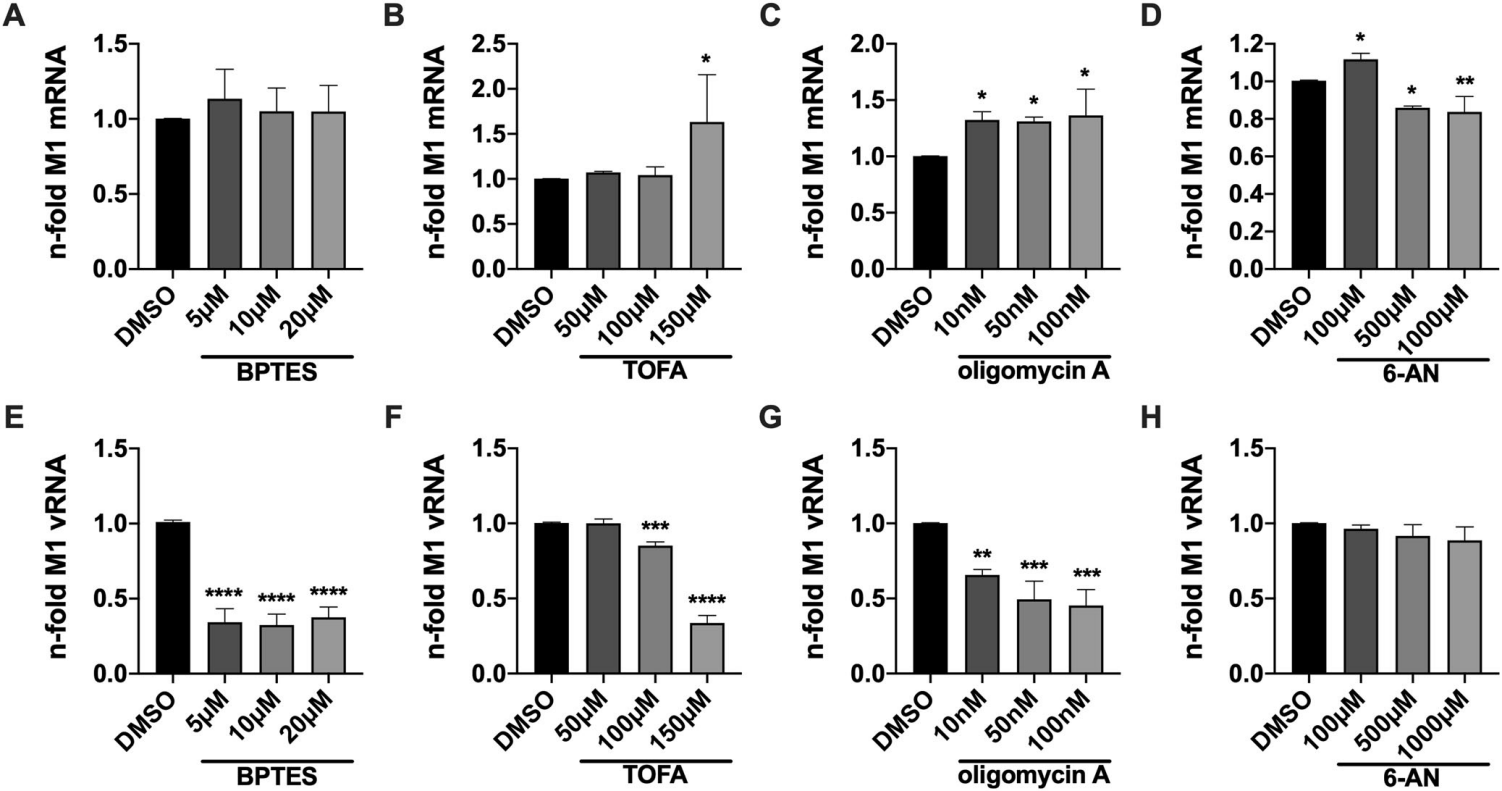
Metabolic interference barely affects viral transcription but reduces replication. A549 cells were infected with SC35M at an MOI of 5 for 30 min. Subsequently, the infected cells were treated with the indicated concentrations of the inhibitors for a total of 8 h since the beginning of the infection. Afterwards, cells were lysed, total RNA was isolated, and cDNA was synthesized using either (**A**–**D**) an oligo(dT) primer to transcribe mRNA or (**E**–**H**) a fluA uni12 primer to transcribe vRNA. **A**–**H** Realtime qPCR was performed with technical duplicates, and the nfolds were calculated in reference to the DMSO control. Depicted are the means ± SD of three independent experiments with biological triplicates per condition and experiment. Statistical significances were determined via ordinary one-way ANOVA with Dunnett’s correction, comparing all treated samples to their DMSO control. *P*-values are indicated as follows: <0.05 = *, <0.01 = **, <0.001 = ***, <0.0001 = ****.

We observed that viral mRNA accumulation was hardly affected within one replication cycle after treatment with any of the four inhibitors (Fig. 4A–D). Upon treatment with higher TOFA concentrations or oligomycin A, there was even a moderate but significant increase of viral mRNA (Fig. 4B–C), and treatment with oligomycin A also led to an increase in viral mRNA amounts (Fig. 4C). 6-AN slightly increased levels of viral mRNA at low concentrations but mildly decreased them at higher concentrations (Fig. 4D). Following these observations, the synthesis or accumulation of vRNA, however, was, depending on the dosage, severely decreased by treatment with three of the four inhibitors (Fig. 4E–H). Only 6-AN did not significantly reduce the accumulation of vRNA within one IAV replication cycle but merely showed a slight trend towards it (Fig. 4H). In general, the patterns of vRNA n-folds 8 hpi resembled those of viral titers after treatment to some degree (Fig. 1I–L), suggesting that metabolic interference mainly impaired the IAV life cycle at the step of vRNA synthesis and thereby reduced viral titers. An exception is the 6-AN mediated drop in viral titers (Fig. 1L), which hardly can be linked to diminished vRNA production within a single replication cycle.

**Fig. 5:**
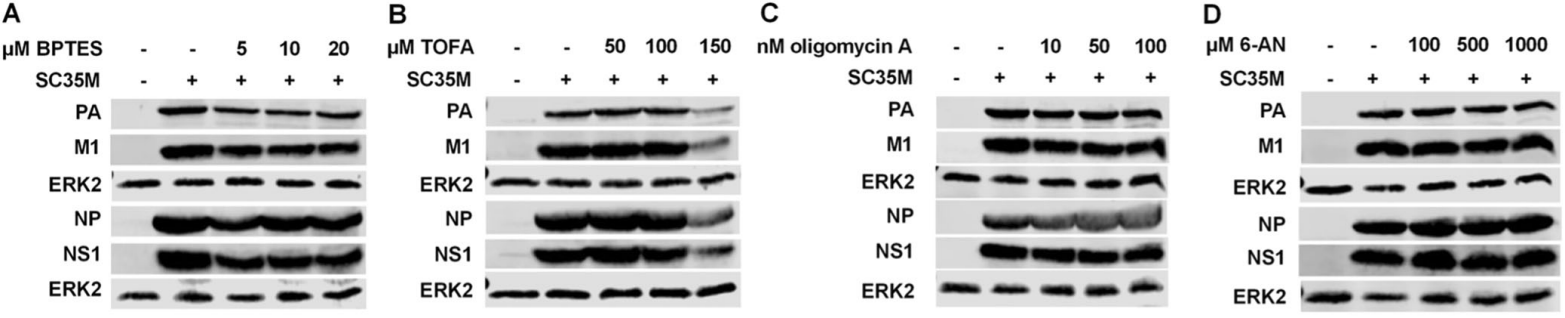
Metabolic interference hardly impairs viral protein synthesis. **A**–**D** A549 cells were infected with SC35M at an MOI of 5 for 30 min. Subsequently, the infected cells were treated with the indicated concentrations of the inhibitors for a total of 8 h since the beginning of the infection. Then, cells were lysed, and proteins were separated via SDS-PAGE. Visualization of protein bands was done using primary antibodies binding PA (rabbit), M1 (mouse), NP (rabbit), NS1 (rabbit) and the loading control ERK2 (rabbit) and fluorescent-labeled anti-mouse (donkey) and anti-rabbit (donkey) secondary antibodies. Illustrated are exemplary protein bands from three independent experiments.

In accordance with these, if any, rather mild effects on the accumulation of viral transcripts, we observed almost no impact of the treatments on viral protein accumulation within a single cycle infection (Fig. 5A–D). The only noteworthy decrease in viral protein accumulation occurred after the treatment with 150 µM TOFA (Fig. 5B), which at the same time led to the strongest increase of viral mRNA (Fig. 4B).

**Fig. 6:**
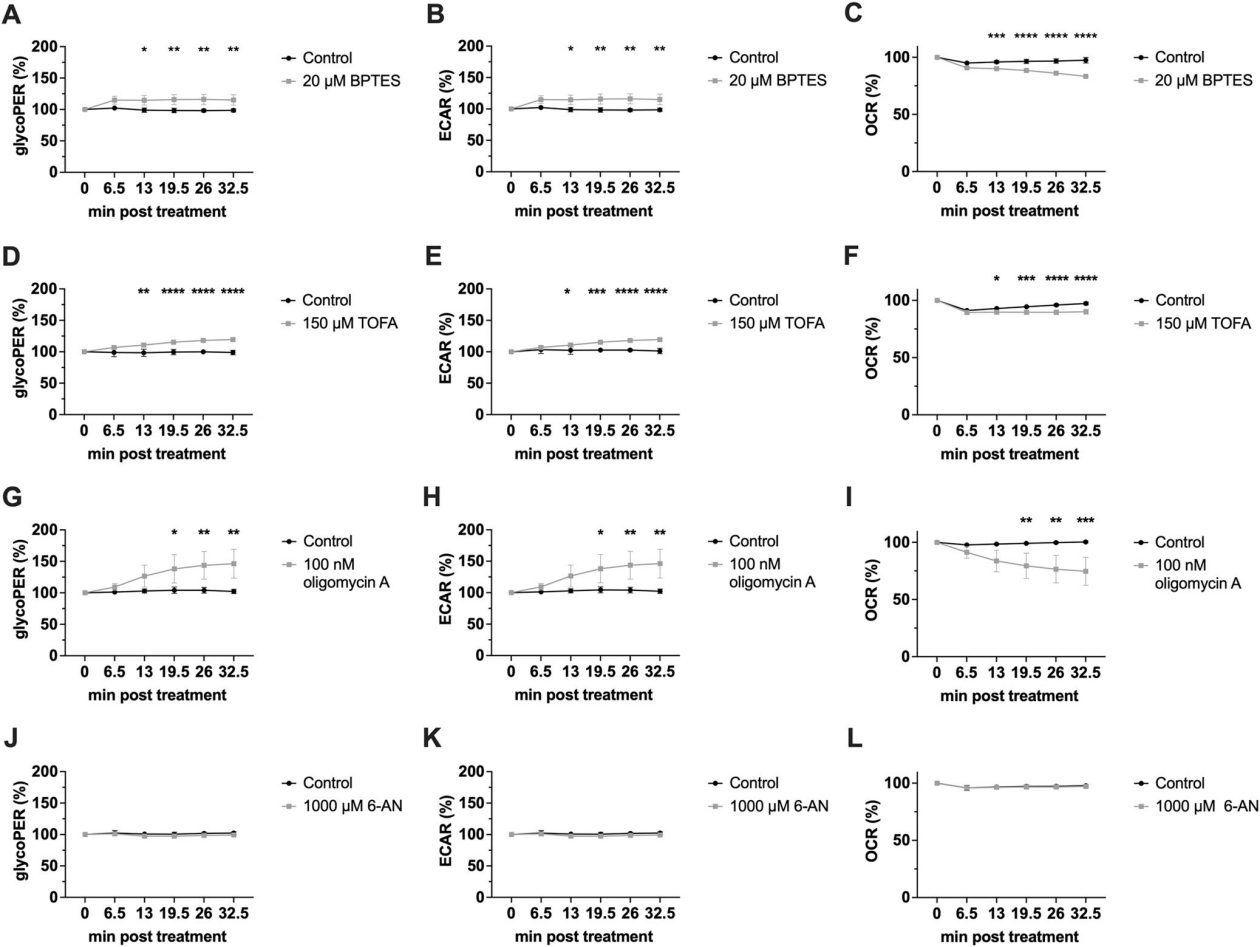
Metabolic interference affects glycolytic rate and cellular respiration. **A–L** 24 h after seeding, A549 cells were incubated in XF DMEM medium supplemented with 25 mM glucose and 2 mM L-glutamine for 1 h at 37 °C in a non-CO_*2*_ incubator. The glycolytic proton efflux rate (glycoPER), the extracellular consumption rate (ECAR), and the oxygen consumption rate (OCR) were measured in real time with a Seahorse Analyzer. Depicted are the means ± SD of three independent experiments with biological triplicates per condition and experiment. Statistical significances were determined via ordinary two-way ANOVA with Sidak’s correction, comparing the treated samples of a time point to its respective DMSO control. *p*-values are indicated as follows: <0.05 = *, <0.01 = **, <0.001 = ***, <0.0001 = ****.

Since we observed decreased viral titers and genomic vRNA levels within one replication cycle upon inhibition of glutaminolysis, FAS, OXPHOS, and the PPP, we aimed to verify the effect on the metabolism during treatment with the four inhibitors. Therefore, we performed cellular energy metabolism measurements in presence or absence of the different metabolic inhibitors in real-time via Seahorse Analyzer. The glycolytic proton efflux rate (glycoPER) and extracellular acidification rate (ECAR) are considered as a direct read-out of the glycolytic rate, whereas the oxygen consumption rate (OCR) is an indicator for cellular respiration. For each of the four inhibitors, we chose the highest previously tested concentration without a cytotoxic effect. Initially, glycoPER, ECAR, and OCR of the samples were measured under basal conditions, followed by injection of the corresponding inhibitors (Fig. 6A–L). Samples treated with 20 µM BPTES or 150 µM TOFA showed a significant increase of glycoPER and ECAR, while the OCR decreased (Fig. 6A–F). Those alterations were moderate but significant and very similar for both treatments. Treatment with 100 nM oligomycin A led to the strongest observed increase of glycoPER and ECAR, while OCR quickly decreased (Fig. 6G–I). In the presence of 6-AN, neither the glycolytic rate nor the cellular respiration rate was significantly altered compared to the DMSO control during the observation period (Fig. 6J–L).

Taken together, treatment of cells with inhibitors for glutaminolysis, FAS and OXPHOS did result in deregulation of the interlinked network of glycolysis and cellular respiration, an observation of interest since it can be connected to the inhibitor-mediated deregulation of IAV polymerase functionalities.

**Fig. 7:**
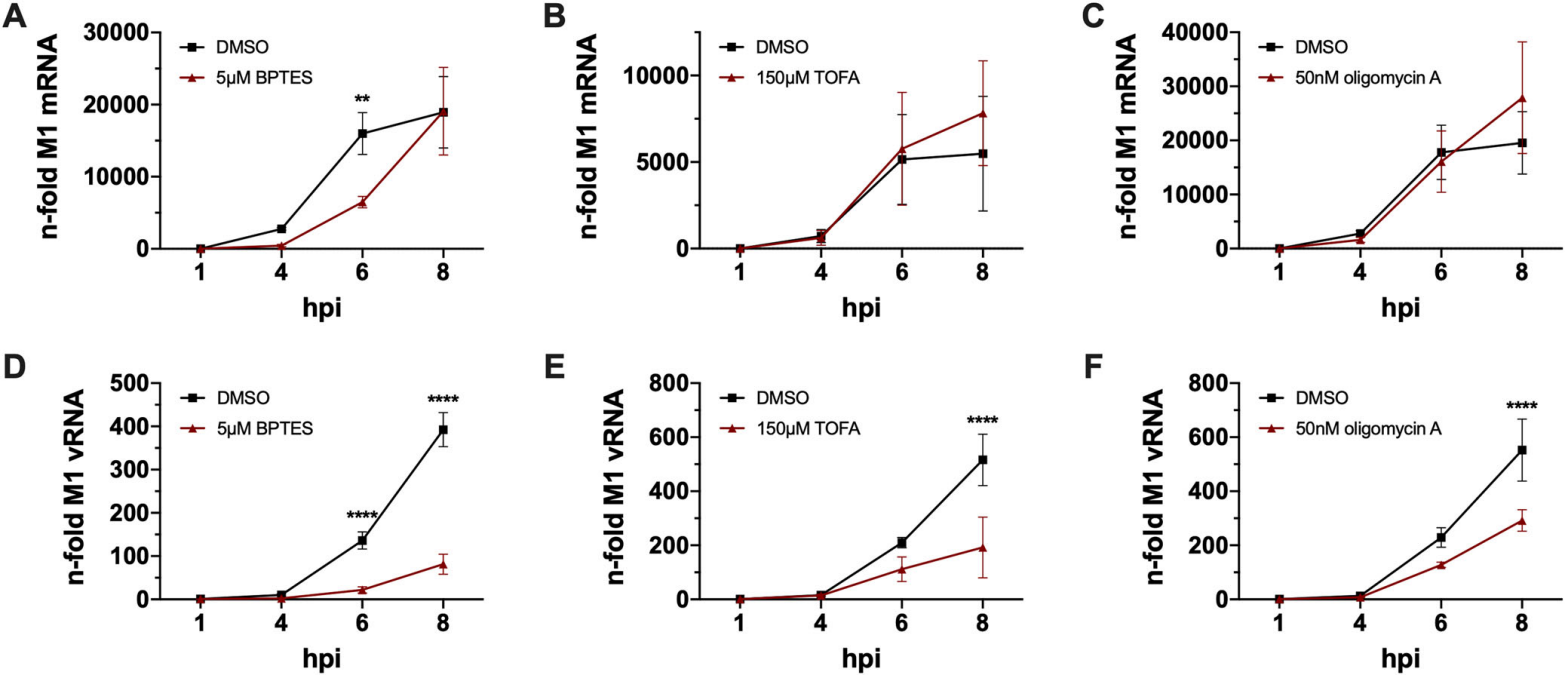
Prolonged mRNA synthesis is accompanied by decreased vRNA synthesis upon metabolic interference. A549 cells were infected with SC35M at an MOI of 5 for 30 min. Subsequently, the infected cells were treated with the indicated concentrations of the inhibitors for a maximum of 8 h since the beginning of the infection. 1, 4, 6 and 8 hpi cells were lysed, total RNA was isolated, and cDNA was synthesized using either (**A**–**C**) an oligo(dT) primer to transcribe mRNA or (**D**–**F**) a fluA uni12 primer to transcribe vRNA. **A**–**F** Realtime qPCR was performed with technical duplicates, and the n-folds were calculated in reference to the 1 h value of the DMSO control. Depicted are the means ± SD of three independent experiments with biological triplicates per condition and experiment. Statistical significances were determined via ordinary two-way ANOVA with Sidak’s correction, comparing the treated sample of a time point to its respective DMSO control. -*P*-values are indicated- as follows: <0.05 = *, <0.01 = **, <0.001 = ***, <0.0001 = ****.

To get a more comprehensive insight into the polymerase-driven processes of transcription and replication, we ran 8 h kinetics and analyzed viral mRNA and vRNA accumulation 1, 4, 6 and 8 hpi in the presence or absence of the metabolic inhibitors. Since 6-AN did not show a significant effect on vRNA synthesis in this scenario, we omitted this experiment with the PPP inhibitor. For the other inhibitors, we chose the concentration that showed the best trade-off between highest antiviral activity and lowest cytotoxicity. Untreated cells usually displayed a strong increase of viral mRNA synthesis until 6 hpi and a subsequent establishment of a plateau, which was illustrated in the black control curves (Fig. 7A–C). Treatment with BPTES led to a reduced mRNA accumulation until the end of the experiment when untreated and treated samples eventually reached the same level of mRNA (Fig. 7A). But from the trend of both curves it can be assumed that mRNA accumulation of BPTES-treated cells would have exceeded that of the DMSO control at later time points. Samples treated with TOFA (Fig. 7B) and oligomycin A (Fig. 7C) showed very similar progression compared to the DMSO controls until 6 hpi. Afterwards, treated cells reached even higher viral mRNA levels than the respective controls. vRNA accumulation, on the other hand, was severely reduced in the presence of all three inhibitors (Fig. 7D–F). This effect significantly increased with progression of time. These kinetics confirmed our previous data shown in Fig. 4 and suggested that the stagnation of viral mRNA levels at about 6 hpi and later in untreated cells did not occur or was delayed in the presence of BPTES, TOFA and oligomycin A and led to a prolonged phase of viral transcription. While viral transcription continued, viral replication was severely decreased, which was marked by lower vRNA levels in treated cells.

Summing up these data, we observed that the applied metabolic inhibitors hardly or, in case of BPTES, only temporarily decreased the synthesis of viral mRNA and also had little effect on viral protein synthesis within a single replication cycle. The spot of intervention within the IAV life cycle seemed to be the synthesis of genomic vRNA, which was strongly decreased after three out of four treatments. Since the viral polymerase was largely able to transcribe mRNA to a normal extent, the kinetics suggested an impairment of the transition of polymerase activity from transcription to replication as the most likely explanation for the observed reductions in vRNA accumulation and viral titers.

To further analyze our phenotype in primary tissue, we used primary human bronchial epithelial cells (HBEpCs) for IAV infections and treatment with BPTES, oligomycin A or TOFA in low concentrations to allow compatibility of the primary tissue. As shown in Supplementary Fig. S2, treatment with BPTES and oligomycin A suppressed production of viral genomic vRNA with no to much weaker effects on viral mRNA levels at an 8 h post-infection point in time in primary human lung cells. For TOFA treatment, we did also observe a much more pronounced reduction of vRNA accumulation, but at this point in time of investigation, viral mRNA accumulation was reduced as well.

Taken together, the data presented here give clear rise to the assumption that the regulation of the viral polymerase to be active as a transcriptase or replicase is regulatory interconnected with the metabolic network of the invaded host cells, opening a new field of research to understand those cross-regulations on the molecular level.

## Discussion

Metabolic intervention might be a promising host-targeted antiviral strategy. However, for such an approach, we must have a detailed understanding of the metabolic host-virus interactions and the dependency of viral replication on the various metabolic branches of the host. Previous publications already showed the profound impact of IAV infections on the host metabolism^5,6,10^. Recently, we demonstrated that inhibition of glycolysis via 2-deoxy-D-glucose (2-DG) severely decreased IAV propagation in a dose-dependent manner. Those data suggested that the treatment with 2-DG interfered with the dynamic regulation of the viral polymerase, determining its function as a transcriptase or replicase. This caused an extended period of viral transcription plus a pronounced reduction of vRNA and, consequently, of viral progeny^10^. Here, we demonstrated that this may be a largely general effect of metabolic interference, mediated by the respective inhibitors potentially due to the high degree of interdependency of metabolic pathways. Inhibition of glutaminolysis, FAS, OXPHOS and the PPP led to significantly decreased viral titers. Moreover, inhibition of glutaminolysis, FAS and OXPHOS resulted in a very similar pattern of affected viral transcription and replication, marked by altered accumulation of mRNA and vRNA, as previously observed after glycolytic inhibition^10^. In line, those three inhibitors showed the potential to critically intervene in the metabolic network of glycolysis and cellular respiration. Therefore, our data suggest a very similar antiviral mechanism as observed upon inhibition of glycolysis, meaning that metabolic interference approaches affecting the glycolysis-respiration balance do impair proper vRNA synthesis by affecting the dynamic regulation of the viral polymerase. Since glutaminolysis, FAS and OXPHOS are all connected to glycolysis, mainly through the TCA cycle, the underlying explanation for the comparable phenotypes regarding viral replication may be overlapping metabolic alterations, mediated by the treatments. Of note, the interlinked network of glycolysis and respiration is differently shifted by using 2-DG compared to treatment with BPTES, TOFA or oligomycin A, but effects on IAV polymerase dysregulation are comparable. Taken together, our data clearly show that unbalancing cellular glycolysis and respiration results in improper regulation of the viral polymerase as a transcriptase or replicase, a fact that needs more research to understand the general impact of the glycolysis-respiration balance on IAV polymerase regulation^10^. Confirming the mechanistic link of misbalanced glycolysis-respiration and IAV polymerase regulation, 6-AN treatment did not affect cellular glycolysis-respiration and did not alter IAV polymerase regulation. As discussed previously^10,11^, the exact regulation of the viral polymerase is still not fully understood, and many viral and host factors are supposed to be involved in its switch from transcription to replication. It is entirely possible that metabolic interference-driven shifting of the glycolysis-respiration balance affects one or several crucial factors which regulate the polymerase’s function and thereby impairs the IAV life cycle. Direct recruitment of metabolic factors to the IAV replication machinery, as seen for the RNA virus tomato bushy stunt virus (TBSV)^12^, could be crucial for IAV polymerase regulation and function and potentially be disturbed by affecting the glycolysis-respiration balance. In addition, direct regulatory effects of certain metabolites are possible since it is already described that various metabolites also have signaling or regulatory functions and can influence gene expression, protein modification, etc^13,14^. One example is lactate, which is not the mere long-considered waste product of glycolysis, but is, among others, involved in regulating lipolysis, immunoregulation and anti-inflammation^15^. Hence, it is possible that one or several metabolites of the aforementioned pathways either have a direct regulatory effect on the viral polymerase or affect it indirectly by influencing the polymerase’s regulatory factors. Identifying these factors and/or potentially involved metabolites as co-factors and unraveling the regulation mechanisms of the viral polymerase in more detail may help to develop more specific treatments. As a general remark, conducting inhibitor-based studies can give rise to questions about certain specificities of treatments. To minimize such thoughts in our study, while setting up this study, we have carefully chosen only well-described and highly characterized inhibitors for installing a high degree of specificity in our metabolic interventions. In addition, using Seahorse-based analyses was very helpful in analyzing specificity and strength of certain metabolic alterations mediated by our chosen inhibitors.

As a final conclusion, more research is needed for fully understanding the molecular interplay of cellular metabolic pathways and even single metabolites or metabolic enzymes and the viral life cycle. Additionally, linking metabolic interventions to potential shiftings in cellular functions and viral activities is critical in this area of research. A step towards a clearer picture of molecular interdependencies between host cell metabolism, cellular functions and the viral life cycle will be achieved by using primary tissue for experiments. Here, we indeed confirmed our observed phenotype of most prominently affected vRNA accumulation after inhibitor treatment in human lung bronchial epithelial cells and thereby further deepened the view on these interactions, clarifying that the viral polymerase regulation to be active as a transcriptase or replicase seems to be regulatory intertwined with the cellular metabolism.

Our data implicate the huge potential of metabolic interference as a host-targeted antiviral strategy. This type of treatment is likely to have a broad antiviral activity and may be considered at least against acute virus infections. Since each virus alters the host metabolism towards its specific requirements, it seems logical that various inhibitors can be combined and adjusted in their concentrations to create virus-tailored antiviral treatment options. However, the reproducibility of our results in vivo and eventually in humans is still pending and would mark very important future milestones.

### Experimental procedures

#### Cell lines and viruses

Human adenocarcinomic alveolar basal epithelial cells (A549, American type culture collection (ATCC®), CCL-185™) and Madin-Darby canine kidney (MDCK) II cells (Institute of Virology, WWU Muenster, Germany) were cultured in Dulbecco’s modified Eagle’s medium high glucose (DMEM, Sigma-Aldrich, D5796) and minimum essential medium (MEM, Sigma-Aldrich, M4655), respectively. Both media were supplemented with 10% fetal bovine serum (FBS). Primary human bronchial epithelial cells (HBEpCs, PromoCell, C-12640) were cultured in airway epithelial cell growth medium (AECGM, PromoCell, C-21060). Cells were incubated at 37 °C and 5% CO_2_. A/Seal/Massachusetts/1/80 H7N7 (SC35M) is a recombinant, mouse-adapted influenza A virus (IAV) strain which was propagated in MDCK II cells. The SC35M virus was chosen for this study due to its high replication capacity and the lack of the need for additional trypsin application under this infection settings to allow the set-up of unbiased virus replication experiments.

#### Infection and inhibitor treatment

The virus stock solution was adjusted to the desired multiplicity of infection (MOI) in phosphate-buffered saline (PBS) supplemented with 0.2% bovine serum albumin (BSA), 1 mM MgCl_2_, 0.9 mM CaCl_2_, 100 U/mL penicillin and 0.1 mg/mL streptomycin. After a washing with PBS and the application of the respective amount of virus, cells were incubated for 30 min at 37 °C and 5% CO_2_. Cells were washed once again and were incubated for the declared periods of time in DMEM (Thermo Fisher Scientific, A14430) containing 0.2% bovine serum albumin (BSA), 100 U/mL penicillin and 0.1 mg/mL streptomycin, 25 mM D-glucose, 2 mM L-glutamine and the respective concentration of inhibitor. Bis-2-(5-phenylacetamido-1,3,4-thiadiazol-2-yl)ethyl sulfide (BPTES, Sigma-Aldrich, SML0601), 5-(tetradecyloxy)-2-furoic acid (TOFA, Cayman Chemical, Cay10005263), oligomycin A (Sigma-Aldrich, 75351) and 6-aminonicotinamide (6-AN, Sigma-Aldrich, A68203) were dissolved in dimethyl sulfoxide (DMSO) to stock concentrations of 10 mM, 75 mM, 50 µM and 500 mM, respectively.

HBEpCs were washed once with PBS after an infection and incubated in AECGM, containing the respective amounts of inhibitors.

#### Plaque titration

After an infection experiment, the number of newly synthesized infectious viral particles was determined as plaque-forming units per milliliter (PFU/mL) as described before^10^.

#### Cytotoxicity assays

Cytotoxic effects of the inhibitors were assessed via two assays. Lactate dehydrogenase (LDH) assays were performed with the CytoSelect LDH cytotoxicity assay kit (Bio Cat, CBA-241-CB) according to the manufacturer’s protocol. For trypan blue exclusions, a 0.4% trypan blue dye (Invitrogen) was mixed 1:1 with a sample’s cell suspension, which was subsequently analyzed by the automated cell counting machine Countess™ II (Invitrogen) to determine the numbers of total and living cells.

#### Reverse transcription and quantitative real-time PCR

Total RNA was isolated using the RNeasy® Plus Mini Kit (QIAGEN) according to the manufacturer’s protocol. RevertAid™ H Minus Reverse Transcriptase (Thermo Fisher Scientific) was used in combination with oligo(dT) primers (Eurofins Genomics) or a fluA uni12 forward primer^16^ (Sigma-Aldrich, 5’-AGCAAAAGCAGG-3’) to synthesize cDNA from mRNA or IAV vRNA, respectively. Real-time qPCR was performed with the LightCycler® 480 II (Roche) and Brilliant III SYBR® Green (Agilent) according to the manufacturer’s instructions. The following primers were used: influenza matrix protein M1 forward (5’-AGA TGA GTC TTC TAA CCG AGG TCG3’) and reverse (5’TGC AAA AAC ATC TTC AAG TCT CTG3’) and human glyceraldehyde 3-phosphate dehydrogenase (GAPDH) forward (5’GCA AAT TCC ATG GCA CCG T3’) and reverse (5’GCC CCA CTT GAT TTT GGA GG3’). GAPDH served as a housekeeping gene for the normalization of mRNA PCR results. The relative n-fold was calculated via the 2^−ΔΔCT^ method^17^.

#### Western blot

Samples were lysed with radioimmunoprecipitation assay (RIPA) buffer (25 mM Tris-HCl pH 8, 137 mM NaCl, 10% glycerol, 0.1% SDS, 0.5% NaDOC, 1% NP-40, 2 mM EDTA pH 8, 200 µM Pefabloc®, 5 µg/mL aprotinin, 5 µg/mL leupeptin, 1 mM sodium orthovanadate and 5 mM benzamidine) at 4 °C. Subsequently, samples were processed as described before^10^. The following primary antibodies were used: ERK2 (rabbit, polyclonal, Santa Cruz, sc-154), M1 (mouse, monoclonal, Biorad, MCA401), NP (rabbit, polyclonal, GeneTex, GTX125989), NS1 (rabbit, polyclonal, GeneTex, GTX125990) and PA (rabbit, polyclonal, GeneTex, GTX125932). ERK2 served as the loading control. Ensuing, fluorophore-labeled secondary antibodies were used: IRDye® 680RD Donkey anti-Mouse (LI-COR, 92668072), IRDye® 680RD Donkey anti-Rabbit (LI-COR, 92668073), IRDye® 800CW Donkey anti-Mouse (LI-COR, 92632212) and IRDye® 800CW Donkey anti-Rabbit (LI-COR, 92632213). Protein bands were visualized with an ODYSSEY® F_C_ Imaging System (LI-COR). Relevant bands were cropped and grouped together. Original blots are shown in Supplementary FigS. S3–10.

#### Measurement of glycolytic rate and cellular respiration

A549 cells were seeded on a Seahorse XFp cell culture miniplate (Agilent) and incubated for 24 h at 37 °C and 5% CO_2_. The Seahorse XFp extracellular flux cartridge (Agilent) was equilibrated with Seahorse XF calibrant solution (Agilent) for 24 h at 37 °C in a non-CO_2_ incubator. The following day, cells were washed with PBS and incubated with Seahorse XF DMEM medium supplemented with 2 mM L-glutamine and 25 mM D-glucose for 1 h at 37 °C in a non-CO_2_ incubator. The ports of the Seahorse XFp extracellular flux cartridge were used for injection of the different metabolic inhibitors BPTES, oligomycin A, TOFA, and 6-AN. Basal levels of extracellular acidification rate (ECAR), oxygen consumption rate (OCR), and glycolytic proton efflux rate (GlycoPER) were determined using the Seahorse XF HS Mini Analyzer (Agilent). After injection of the indicated concentrations of the inhibitors ECAR, OCR, and glycoPER were measured for a total of 1 h.

## Supplementary information

Supplementary information

## Data Availability

All relevant data are within the paper and its Supplementary material files.
