# Supplementary material for: Metabolic interference impairs influenza A virus replication by dampening vRNA synthesis
Source: Npj Viruses. 2025 Mar 28;3:22. doi: 10.1038/s44298-025-00090-4 (PMC11953304; doi:10.1038/s44298-025-00090-4)
Supplement: Supplementary file 1 — Supplementary information [file 44298_2025_90_MOESM1_ESM.docx]

**Supplementary**

**
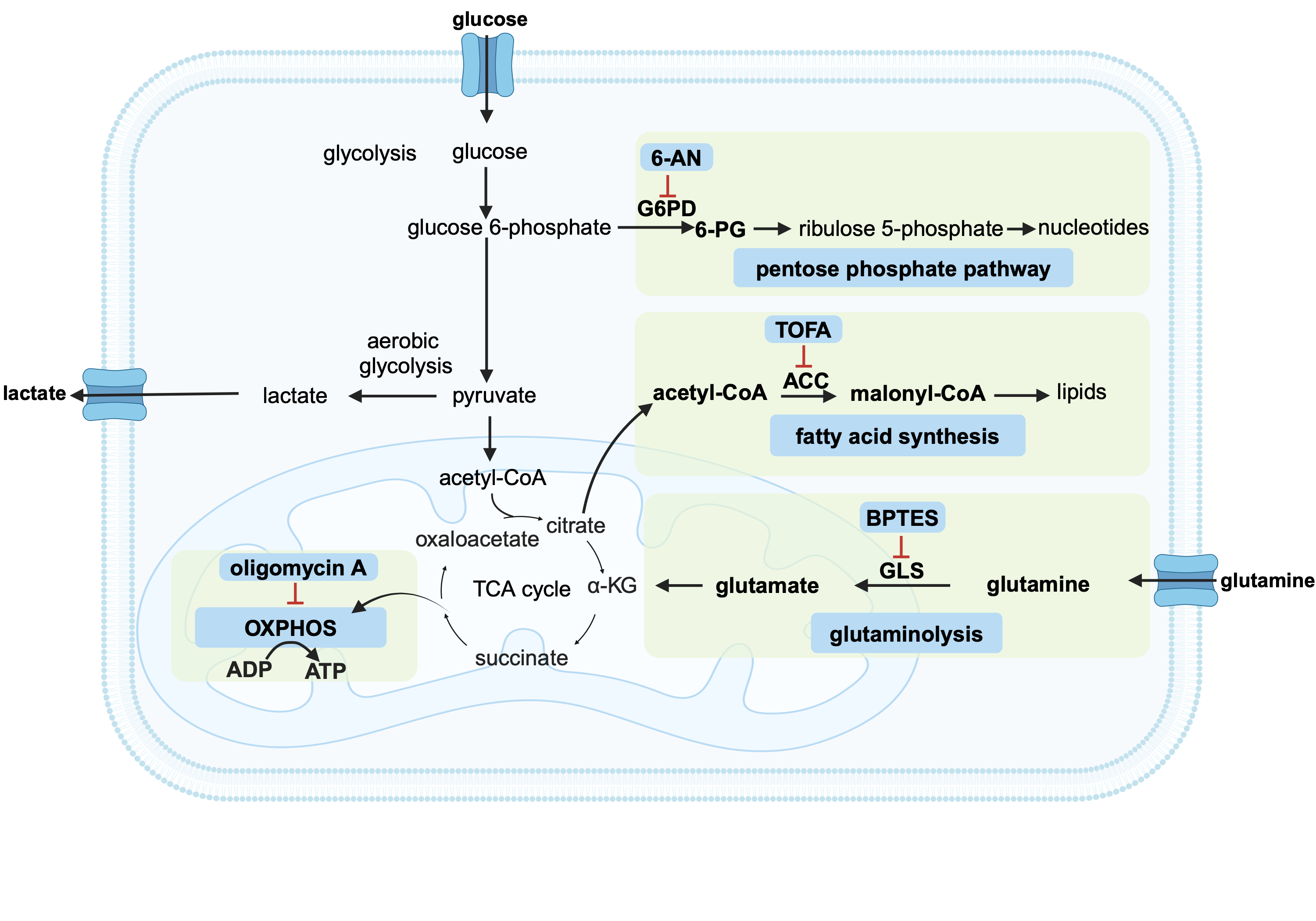
**

**Fig S1: Schematic representation of main metabolic pathways and their specific inhibitors.**The graphic illustrates central metabolic pathways and highlights points of metabolic intervention by different inhibitors. The pentose phosphate pathway (PPP) inhibitor 6-aminonicotinamide (6-AN) targets the NADP^+^-dependent enzyme glucose 6-phosphate dehydrogenase (G6PD). 5-(tetradecyloxy)-2-furoic acid (TOFA) is an inhibitor of acetyl-CoA carboxylase (ACC), an essential enzyme involved in the fatty acid synthesis. Bis-2-(5-phenylacetamido-1,3,4-thiadiazol-2-yl)ethyl sulfide (BPTES) blocks glutaminase (GLS), reducing glutaminolysis activity. Oxidative phosphorylation (OXPHOS) is inhibited by oligomycin A, which acts as a mitochondrial ATP synthase inhibitor. The schematic illustration was created with BioRender.com.


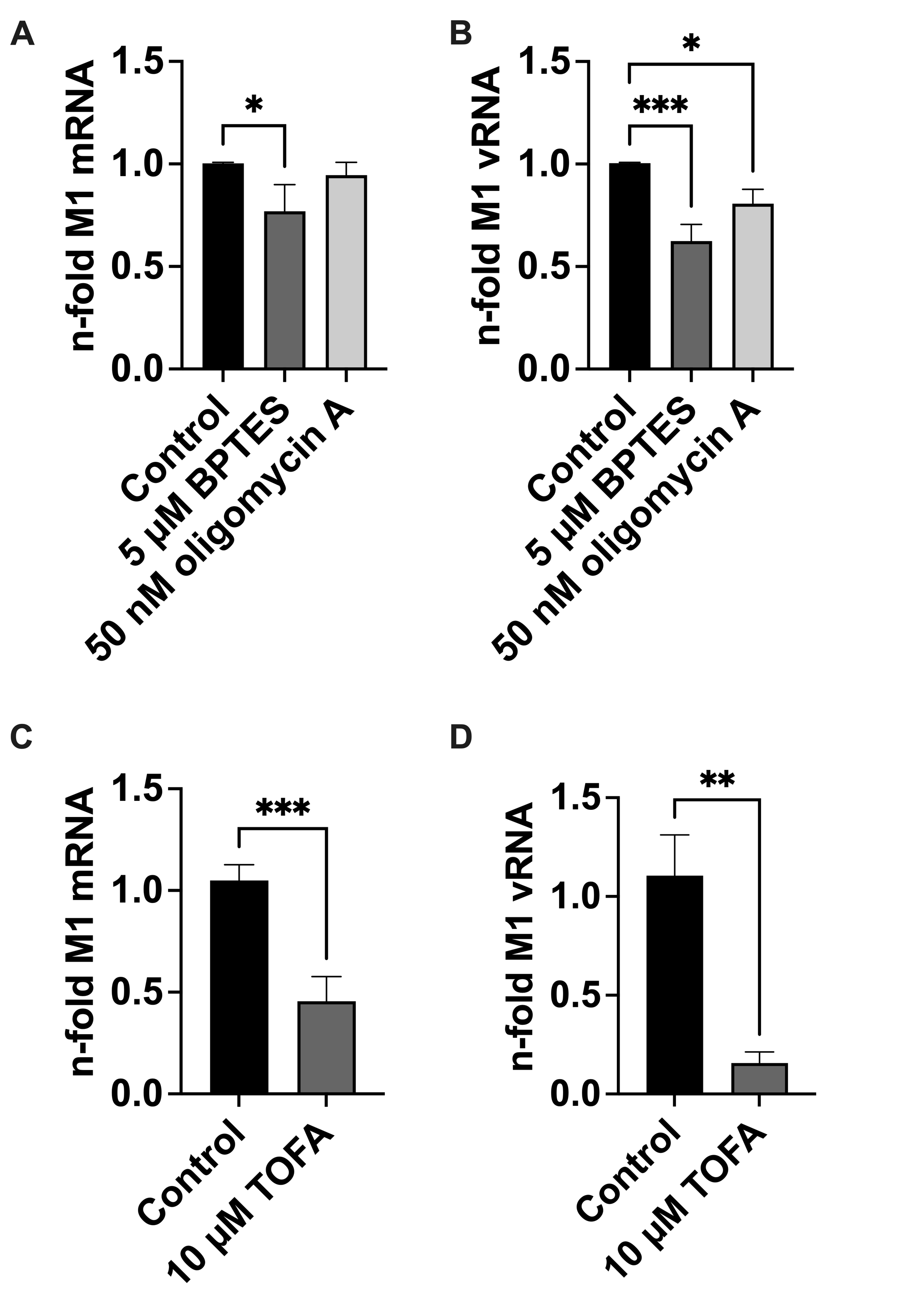


**Fig S*2*: Metabolic interference primarily affects viral genomic RNA (vRNA) accumulation in primary human lung cells.**Primary human bronchial epithelial cells (HBEpCs) were infected with SC35M at an MOI of 5 for 30 min and were incubated with the indicated concentrations of the inhibitors for a total of 8 h since the beginning of infection. Subsequently, cells were lysed, their RNA isolated and cDNA synthesized using either (**A+C**) oligo (dt) primers to transcribe mRNA or (**B+D**) flu uni12 primers to transcribe vRNA. Real-time qPCR was performed with two technical replicates per sample and the n-folds were calculated in reference to the DMSO control. Depicted are the means ± SD of ***(A+B)*** three and ***(C+D)*** four independent experiments with biological triplicates per condition and experiment. Statistical significances were determined (**A+B**) via ordinary one-way ANOVA with Dunnett’s correction, or ***(*C+D*)*** via unpaired Welch’s t test comparing all treated samples to their DMSO control. P-values are indicated as follows: < 0.05 = *, < 0.01 = **, < 0.001 = ***, < 0.0001 = ****.


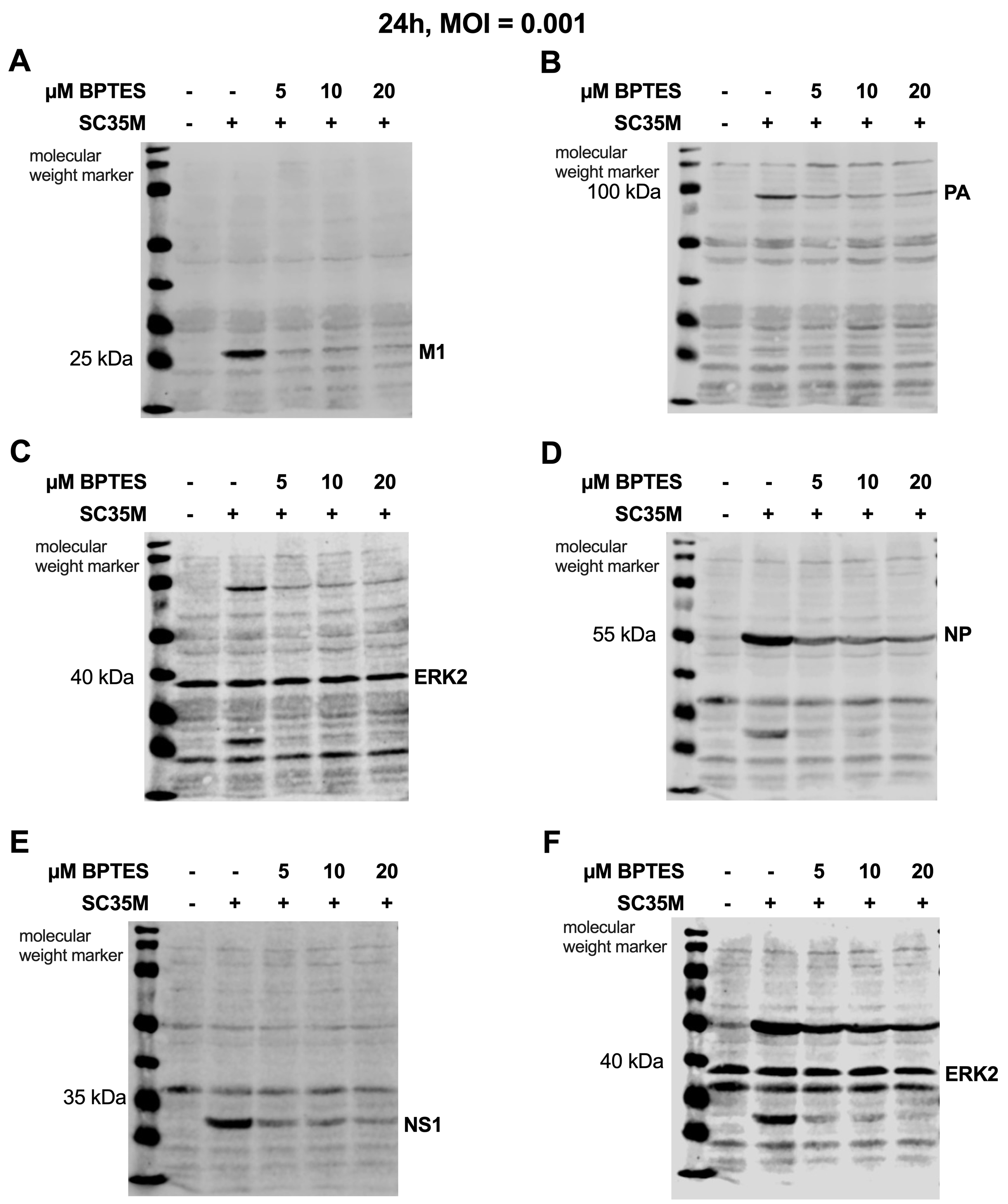


**Fig S3: Corresponding blots of Fig 2 with BPTES treatment.** A549 cells were infected with SC35M at an MOI of 0.001 for 30 min. Subsequently, the infected cells were treated with the indicated concentrations of the inhibitors for a total of 24 h since the beginning of the infection. Then cells were lysed and proteins were separated via SDS-PAGE. Visualization of protein bands was done using primary antibodies binding PA (rabbit), M1 (mouse), NP (rabbit), NS1 (rabbit) and the loading control ERK2 (rabbit) and fluorescent-labelled anti-mouse (donkey) and anti-rabbit (donkey) secondary antibodies. Illustrated are all original blots used for Fig. 2 BPTES treatment.


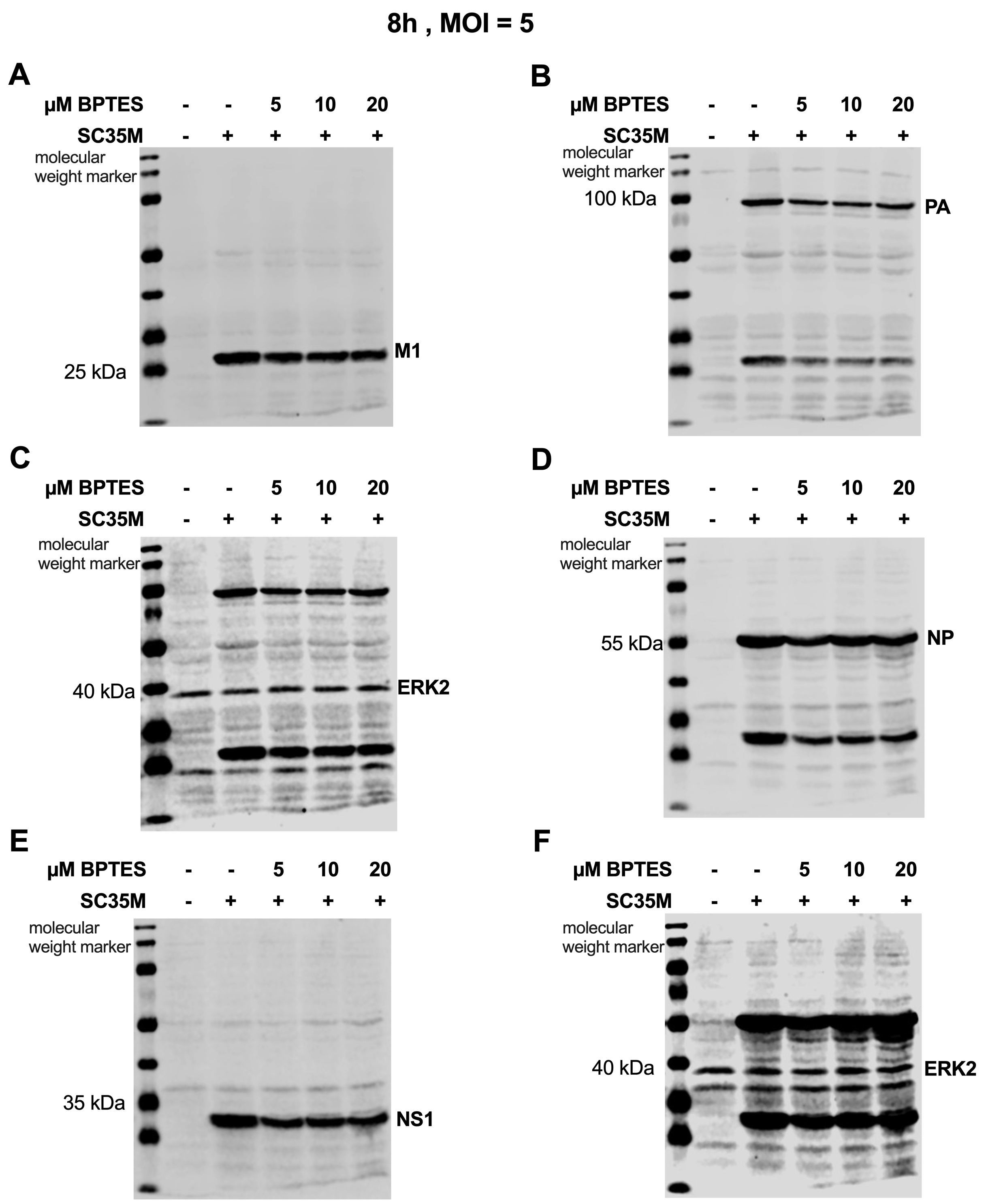


**Fig S4: Corresponding blots of Fig 5 with BPTES treatment.** A549 cells were infected with SC35M at an MOI of 5 for 30 min. Subsequently, the infected cells were treated with the indicated concentrations of the inhibitors for a total of 8 h since the beginning of the infection. Then cells were lysed and proteins were separated via SDS-PAGE. Visualization of protein bands was done using primary antibodies binding PA (rabbit), M1 (mouse), NP (rabbit), NS1 (rabbit) and the loading control ERK2 (rabbit) and fluorescent-labelled anti-mouse (donkey) and anti-rabbit (donkey) secondary antibodies. Illustrated are all original blots used for Fig. 5 BPTES treatment.

**
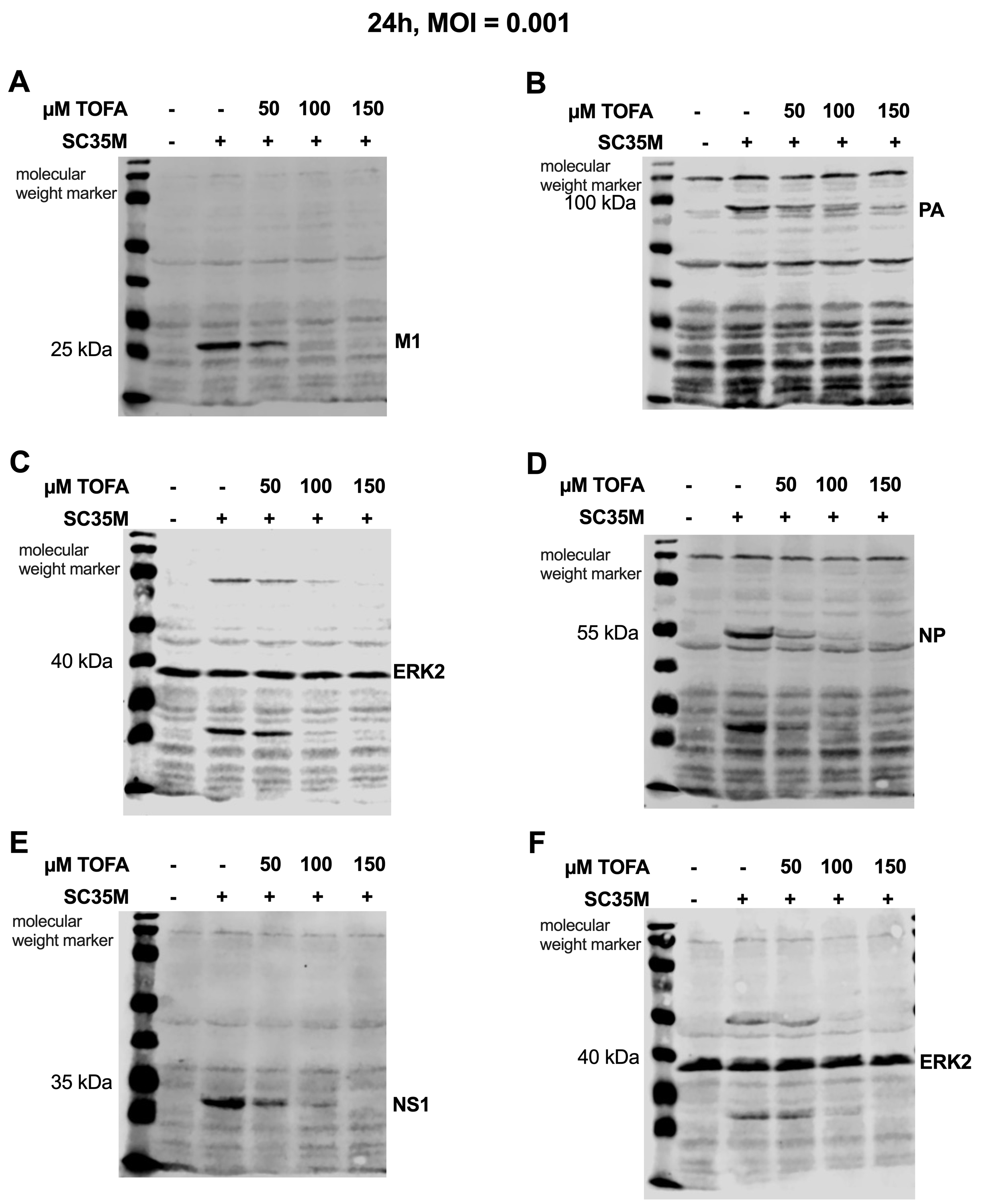
**

**Fig S5: Corresponding blots of Fig 2 with TOFA treatment.** A549 cells were infected with SC35M at an MOI of 0.001 for 30 min. Subsequently, the infected cells were treated with the indicated concentrations of the inhibitors for a total of 24 h since the beginning of the infection. Then cells were lysed and proteins were separated via SDS-PAGE. Visualization of protein bands was done using primary antibodies binding PA (rabbit), M1 (mouse), NP (rabbit), NS1 (rabbit) and the loading control ERK2 (rabbit) and fluorescent-labelled anti-mouse (donkey) and anti-rabbit (donkey) secondary antibodies. Illustrated are all original blots used for Fig. 2 TOFA treatment.

**
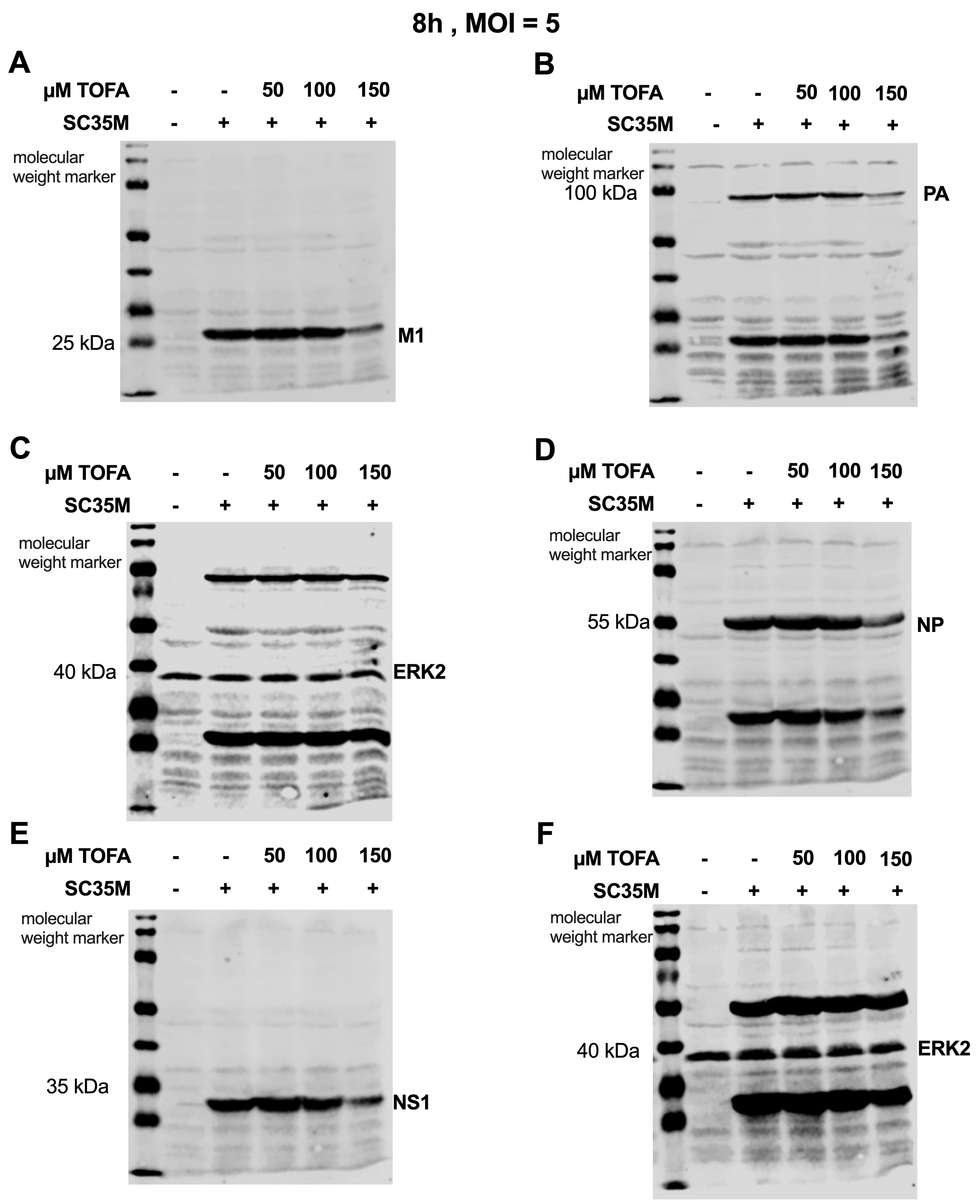
**

**Fig S6: Corresponding blots of Fig 5 with TOFA treatment.** A549 cells were infected with SC35M at an MOI of 5 for 30 min. Subsequently, the infected cells were treated with the indicated concentrations of the inhibitors for a total of 8 h since the beginning of the infection. Then cells were lysed and proteins were separated via SDS-PAGE. Visualization of protein bands was done using primary antibodies binding PA (rabbit), M1 (mouse), NP (rabbit), NS1 (rabbit) and the loading control ERK2 (rabbit) and fluorescent-labelled anti-mouse (donkey) and anti-rabbit (donkey) secondary antibodies. Illustrated are all original blots use for Fig. 5 TOFA treatment.

**
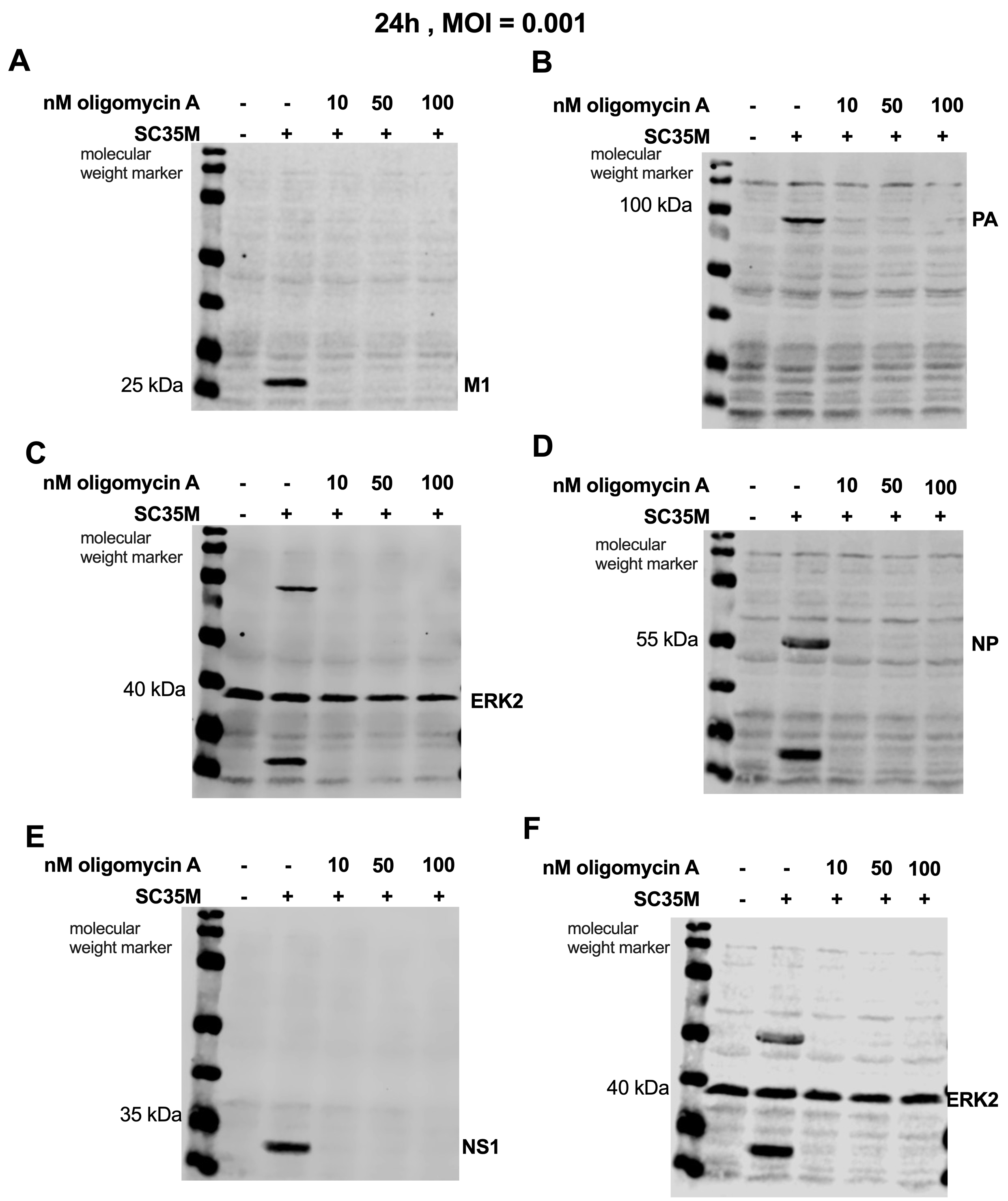
**

**Fig S7: Corresponding blots of Fig 2 with oligomycin A treatment.** A549 cells were infected with SC35M at an MOI of 5 for 30 min. Subsequently, the infected cells were treated with the indicated concentrations of the inhibitors for a total of 8 h since the beginning of the infection. Then cells were lysed and proteins were separated via SDS-PAGE. Visualization of protein bands was done using primary antibodies binding PA (rabbit), M1 (mouse), NP (rabbit), NS1 (rabbit) and the loading control ERK2 (rabbit) and fluorescent-labelled anti-mouse (donkey) and anti-rabbit (donkey) secondary antibodies. Illustrated are all original blots used for Fig. 2 oligomycin A treatment.

**
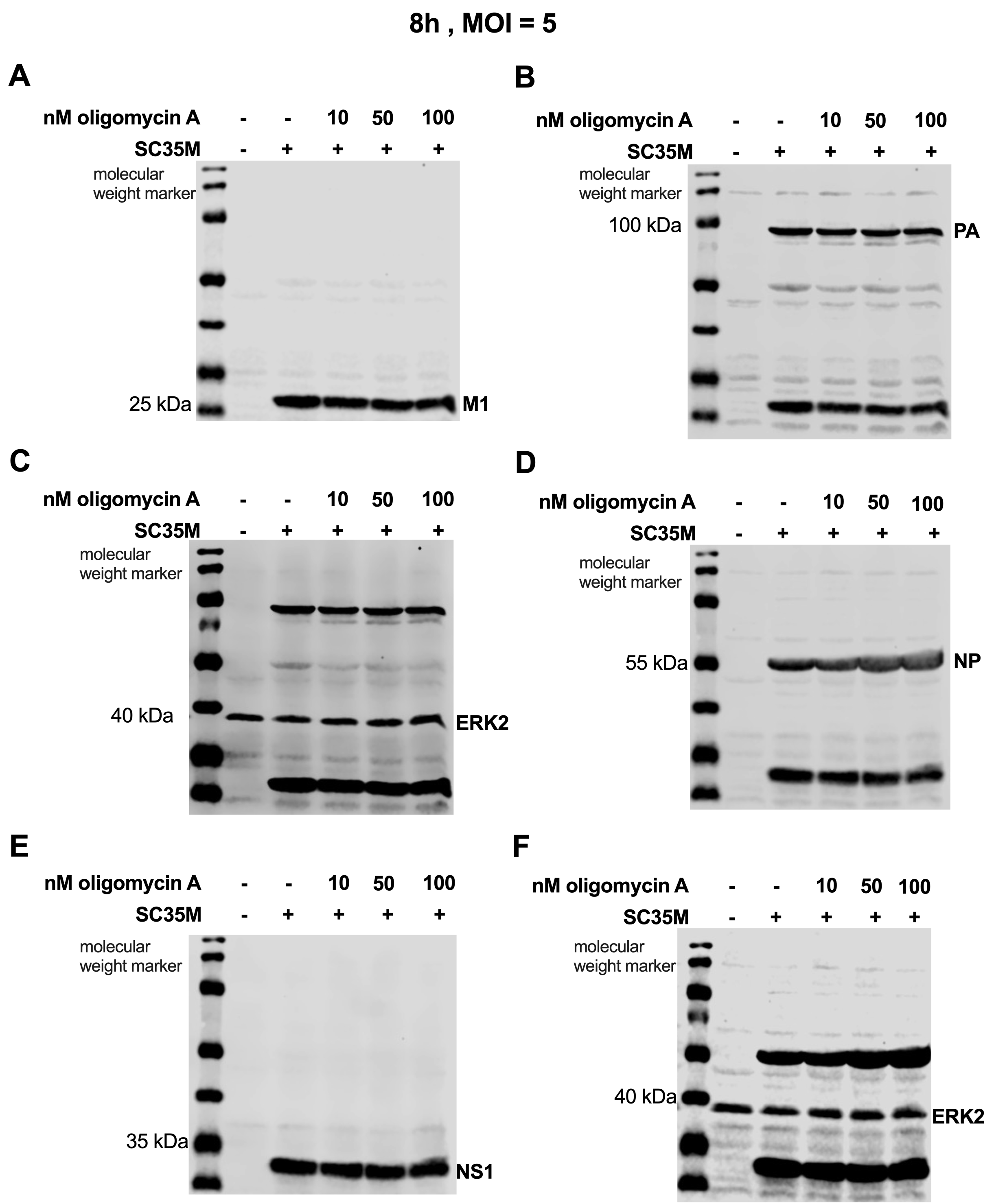
 Fig S8: Corresponding blots of Fig 5 with oligomycin A treatment.** A549 cells were infected with SC35M at an MOI of 5 for 30 min. Subsequently, the infected cells were treated with the indicated concentrations of the inhibitors for a total of 8 h since the beginning of the infection. Then cells were lysed and proteins were separated via SDS-PAGE. Visualization of protein bands was done using primary antibodies binding PA (rabbit), M1 (mouse), NP (rabbit), NS1 (rabbit) and the loading control ERK2 (rabbit) and fluorescent-labelled anti-mouse (donkey) and anti-rabbit (donkey) secondary antibodies. Illustrated are all original blots used for Fig. 5 oligomycin A treatment.

**
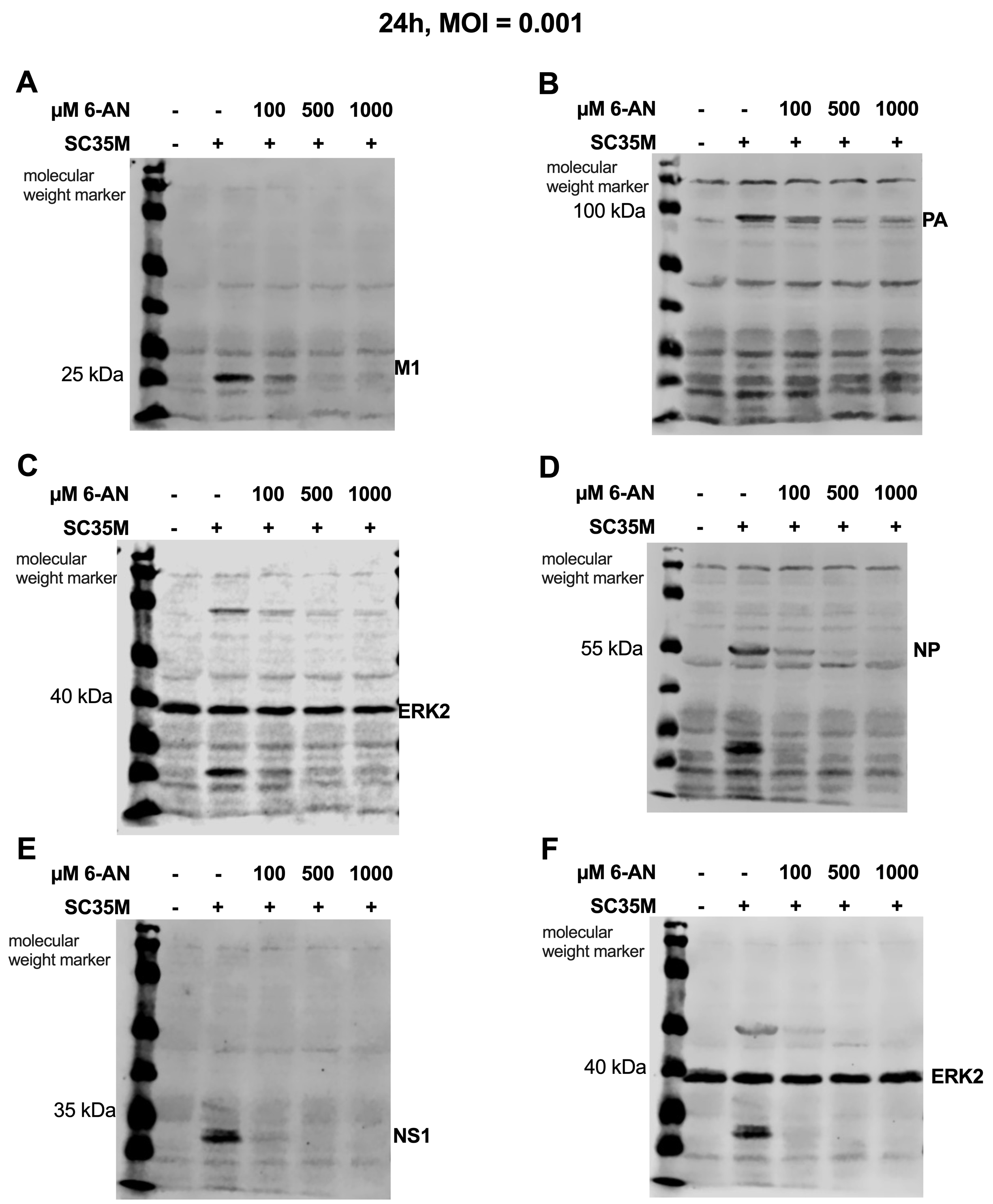
**

**Fig S9: Corresponding blots of Fig 2 with 6-AN treatment.** A549 cells were infected with SC35M at an MOI of 0.001 for 30 min. Subsequently, the infected cells were treated with the indicated concentrations of the inhibitors for a total of 24 h since the beginning of the infection. Then cells were lysed and proteins were separated via SDS-PAGE. Visualization of protein bands was done using primary antibodies binding PA (rabbit), M1 (mouse), NP (rabbit), NS1 (rabbit) and the loading control ERK2 (rabbit) and fluorescent-labelled anti-mouse (donkey) and anti-rabbit (donkey) secondary antibodies. Illustrated are all original blots used for Fig. 2 6-AN treatment.


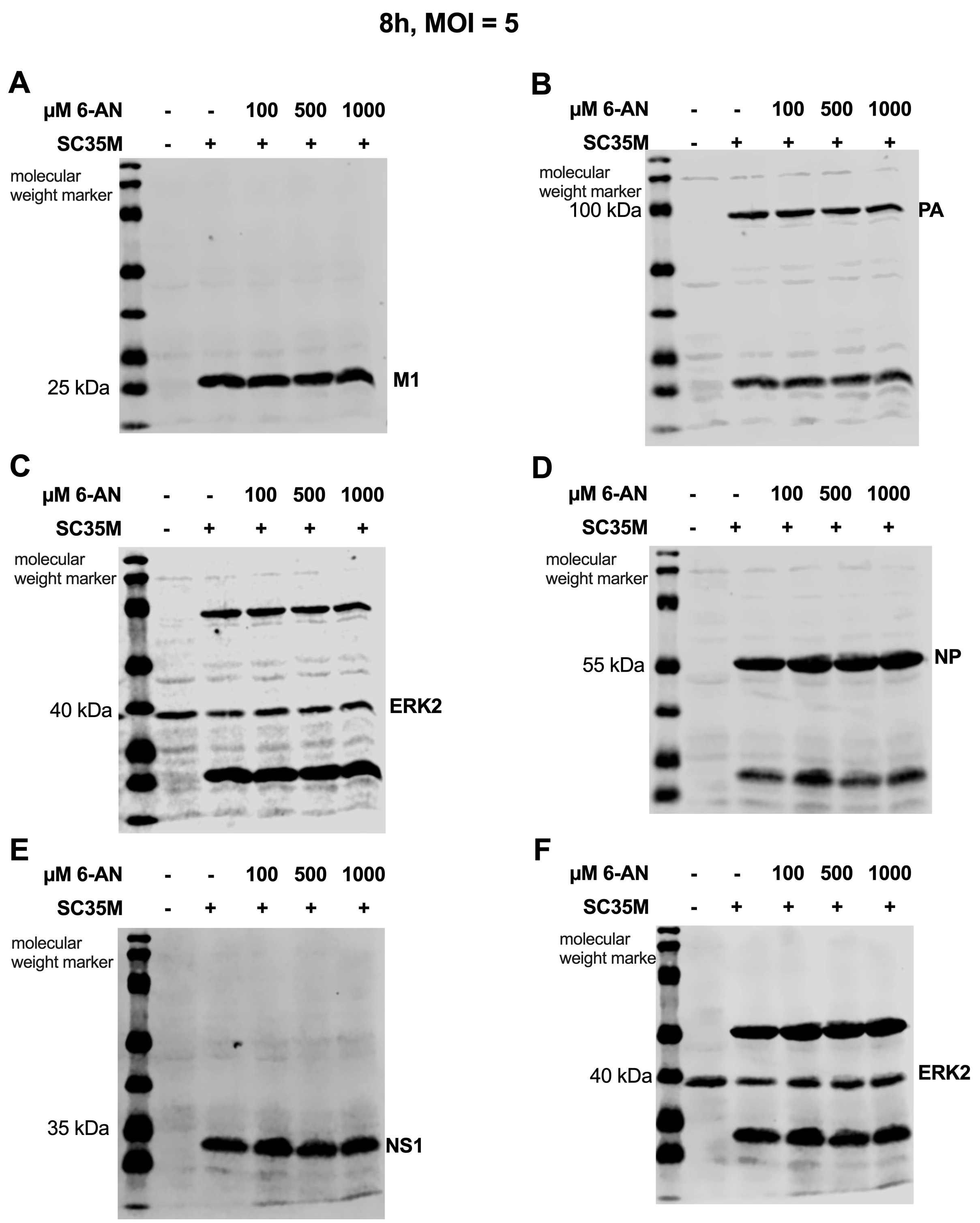


**Fig S10: Corresponding blots of Fig 5 with 6-AN treatment.** A549 cells were infected with SC35M at an MOI of 5 for 30 min. Subsequently, the infected cells were treated with the indicated concentrations of the inhibitors for a total of 8 h since the beginning of the infection. Then cells were lysed and proteins were separated via SDS-PAGE. Visualization of protein bands was done using primary antibodies binding PA (rabbit), M1 (mouse), NP (rabbit), NS1 (rabbit) and the loading control ERK2 (rabbit) and fluorescent-labelled anti-mouse (donkey) and anti-rabbit (donkey) secondary antibodies. Illustrated are all original blots used for Fig. 5 6-AN treatment.
